# Elevated homocysteine, as a biomarker of cardiac injury, in panic disorder patients due to oxidative stress

**DOI:** 10.1002/brb3.1851

**Published:** 2020-09-23

**Authors:** Seyed Shahrokh Aghayan, Asghar Farajzadeh, Zahra Bagheri‐Hosseinabadi, Homeyra Fadaei, Maryam Yarmohammadi, Moslem Jafarisani

**Affiliations:** ^1^ School of Medicine Shahroud University of Medical Sciences Shahroud Iran; ^2^ Clinical Biochemistry Islamic Azad University Ardabil Iran; ^3^ Department of Clinical Biochemistry School of Medicine Rafsanjan University of Medical Sciences Rafsanjan Iran; ^4^ Department of Medical sciences Babol Branch Islamic Azad University Babol Iran; ^5^ Clinical Biochemistry School of Medicine Shahroud University of Medical Sciences Shahroud Iran

**Keywords:** carbonyl groups, glutathione, homocysteine, panic disorder

## Abstract

**Background and Objective:**

Patients with panic disorder (PD) suffer from elevated oxidative stress as a consequence of serotonin metabolism disorder. These patients have elevated serotonin concentration and catabolism of serotonin via monoamine oxidase. The aim of the present study was to evaluate serum homocysteine concentration and its relationship with oxidative stress level in PD patients, regarding homocysteine as a diagnostic biomarker of heart disease.

**Materials & Method:**

Sixty patients with PD according to the DSM‐5 diagnostic criteria for a panic attack and 60 healthy individuals were included in the present study.

Peripheral venous blood samples were taken from patients. Erythrocytes and serum were separated from blood, and RBC hemolysates were prepared to investigate oxidative stress indices including glutathione and glutathione peroxidase. Serum homocysteine and carbonyl groups concentrations were measured in all samples. Data were analyzed using ANOVA, and *p* < .05 was considered significant.

**Results:**

Results showed that serum carbonyl groups concentration was significantly higher in patients with PD than in healthy individuals (*p* < .001). The results also indicated decreased serum glutathione concentration and glutathione peroxidase activity in patients (*p* < .003). In addition, elevated homocysteine concentration in PD patients serum was observed during the present study (*p* < .003).

**Conclusion:**

Our findings support that patients with PD experience higher levels of oxidative stress, due to impaired serotonin metabolism, which is related to the prognosis of heart disease in these patients.

## Graphical abstract

The aim of the present study was to evaluate serum homocysteine concentration and its relationship with oxidative stress levels in patients with PD, regarding homocysteine as a diagnostic biomarker of heart disease. Our findings support that, patients with PD experience higher levels of oxidative stress, due to impaired serotonin metabolism, which is related to the prognosis of heart disease in these patients.

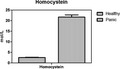

## Significant Outcome

- Current data in Panic disorder (PD) patients notified increase in oxidative stress due to serotonin metabolism impairment.
- Homocysteine promise as biomarkers for cardiovascular disorder in PD patients.

## Limitation

- The study may not have enough power to detect serotonin metabolites.
- The lack of validation experiments is a caveat.

## 1 INTRODUCTION

Homocysteine is a neurotoxic and sulfuric amino acid that is rapidly picked up by neurons via a specific transporter at the membrane surface (Mattson & Shea, 2003). Studies link elevated plasma levels of homocysteine to increased risk of cardiovascular disease (Welch & Loscalzo, 1998). First, the association between elevated plasma homocysteine level and the risk of heart disease was reported in patient with deficiency or absence of cystathionine synthase (Mudd et al., 1964), and further studies confirmed this association (Boushey et al., 1995; Nygård et al., 1997). Proposed mechanisms for the association between elevated levels of plasma homocysteine and cardiovascular disease include impaired endothelial function, increased levels of oxidized LDL, proliferation of vascular Smooth cells, and impaired coagulation processes.

Studies have shown links between heart disease and psychological illness such as anxiety, depression, and panic (Fleet et al., 2000; Haines et al., 1987; Hemingway & Marmot, 1999; Kawachi et al., 1994). However, they do not emphasize anxiety or panic as the cause of cardiovascular disorders (Kawachi, et al., 1994; Shen et al., 2008). There have been reports of decreased heart rhythm and panic or anxiety that suggested decrease in vagal tone and increase in sympathetic nerve as cause of the disease (Kawachi et al., 1995; Yeragani et al., 1990, 1993). Further, elevated plasma levels of homocysteine have been reported in neurodegenerative diseases such as Alzheimer's, schizophrenia, and depression (Bottiglieri et al., 2000; Levine et al., 2001; Seshadri et al., 2002).

A significant association between elevated plasma homocysteine levels and depression has been reported by a number of researchers, which predicted that more than half of those with depression will experience elevated plasma homocysteine levels (Bottiglieri et al., 2000; Reif et al., 2003; Tiemeier et al., 2002). In addition, the association between homocysteine and stress in human and animal models has been reported (Kang et al., 2005; de Oliveira et al., 2004; de Souza et al., 2006).

Panic disorder (PD) is a chronic debilitating neurodegenerative disease that affects about 1.8% of the populations (Craske et al., 2010). Although the pathogenesis of the disease is not fully understood, various studies reported impaired serotonin metabolism in these patients (Zangrossi, 2020). Increased level of serotonin elevates its catabolism and activity of monoamine oxidase (Floris et al., 2020). Selective serotonin reuptake inhibitors, used in the treatment of panic patients, elevate serotonin level and catabolism (Quagliato et al., 2019). Various studies have shown that increased monoamine oxidase activity induces oxidative stress, which causes inflammation and cardiovascular disease (Gupta et al., 2019; Sturza, et al., 2019; Sturza, et al., 2019; Xu et al., 2019). Since serotonin metabolism abnormalities and increased monoamine oxidase activity have been reported in panic patients, the aim of present study was to evaluate homocysteine levels as a biomarker of heart disease in panic patients.

## 2 METHODS

In the present case–control study, 60 patients with PD diagnosed by neuropsychiatric and 60 healthy individuals were investigated. The patients were standardized according to the PD diagnosis guideline DSM‐5 diagnostic criteria for a panic attack. The study was conducted under the Code of Ethics IR.SHMU.REC.1397.90 approved by the Ethics Committee of Shahroud University of Medical Sciences. All subjects included using a signed informed consent form. Patients with a history of heart disease; diabetes, chronic liver or kidney disease, and tobacco use were excluded from the study. The groups were matched for sex and age.

### 2.1 Sample preparation

Five ml of fasting venous blood was collected from all the 120 subjects. One ml of specimens was transferred to EDTA‐containing tubes, and the rest were moved to the laboratory for serum separation.

### 2.2 Red blood cell glutathione and glutathione peroxidase assays

Commercial GSH assay kit (Oxford Biomedical Research Inc.) was used for evaluation of Red blood cell glutathione level and glutathione peroxidase activity. Manufacturer's instructions were strictly followed during the assay. Five hundred micro litre of whole blood was mixed with 500 µl of deionized water and hemolysates, and the GSH/GSSG ratio was calculated. Hemolysates of previous step were used to measure GPX of erythrocytes. Complied with the Cayman's Glutathione Peroxidase Assay Kit guide and achieved GPX activity.

### 2.3 Measurement of carbonyl groups

Serum carbonyl groups were measured by dinitrophenylhydrazine (DNPH) method (Levine et al., 1990). Briefly, a protein suspension from each sample was prepared by adding 1 ml of 10% trichloroacetic acid to 200 µl of serum and centrifuged at 600 *g* at 4°C for 5 min. The supernatant was discarded, and the precipitate was dissolved in 1 ml of HCl 2 M and DNPH 0.2% and incubated at room temperature for 1 hr. Then, the proteins were precipitated with cold TCA 10% at 1,200 *g* for 10 min. The precipitates were washed twice with 1:1 ethanol: ethyl acetate solution and dissolved in 1.5 ml of 20 Na_3_PO_4_ mM at pH6.5. Next, the solution of guanidine hydrochloride 6 mM was added, and the optical absorption of solution at 370 nm was measured by Shimadzu spectrophotometer to detect carbonyl protein group.

### 2.4 Homocysteine assay

Homocysteine levels were measured by HPLC method (Araki & Sako, 1987). For this purpose, samples were collected in cold ethylene diaminotetracetic acid and then serum was separated by centrifugation.

### 2.5 Statistical analysis

The data were expressed as mean ± *SD*, and *T* test was used for statistical analysis. The statistical significance level was considered *p* < .05.

## 3 RESULTS

The mean age of the study population was 34.38 ± 11.23 years. Of the total studied subjects (120), 69 were female (57.5%) and the rest were male (42.5%). No significant relationship was found between age and sex and disease (Table 1).

**Table 1:** Demographic characteristics of panic disorder patients and healthy individuals

Variable	Mean ± Standard deviation	
Age (year)	34.38 ± 11.23	
Panic patients	32.25 ± 10.45	
Healthy individuals	35.14 ± 9.65	
Average age since diagnostic	5.23 ± 2.12	
Gender (%)		Age (year)
Male	51 (42.5)	38.21 ± 9.41
Female	69 (57.5)	29.36 ± 8.45

Note

Based on nonpaired *t* test and the Fisher test, no significant correlation was found.

**Figure 1:**
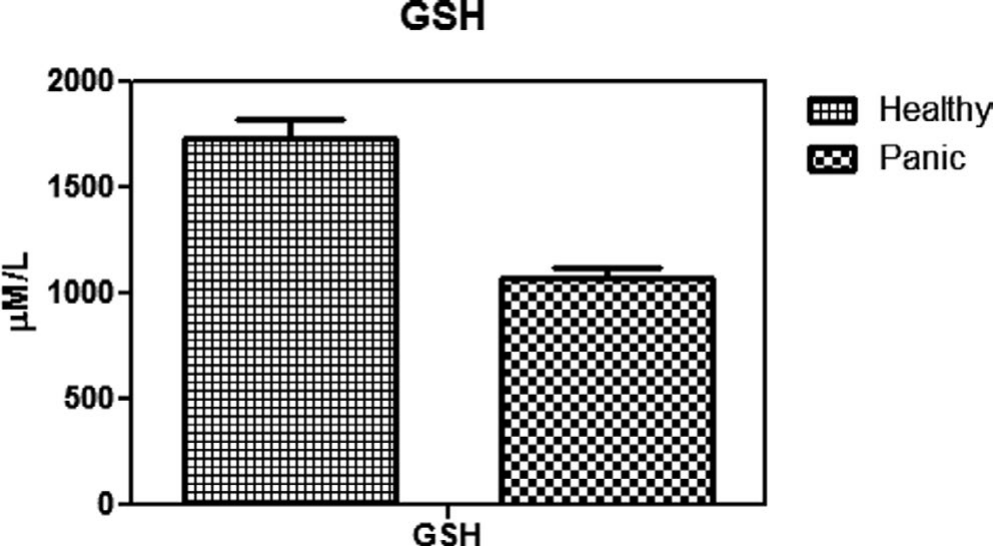
Comparison of GSH between Healthy individuals and panic disorder (PD) patients. It showed GSH concentration has a significant decrease in PD patients. PD patients have lower levels of red blood cell glutathione than healthy people, which was statistically significant (*p* < .003)

**Figure 2:**
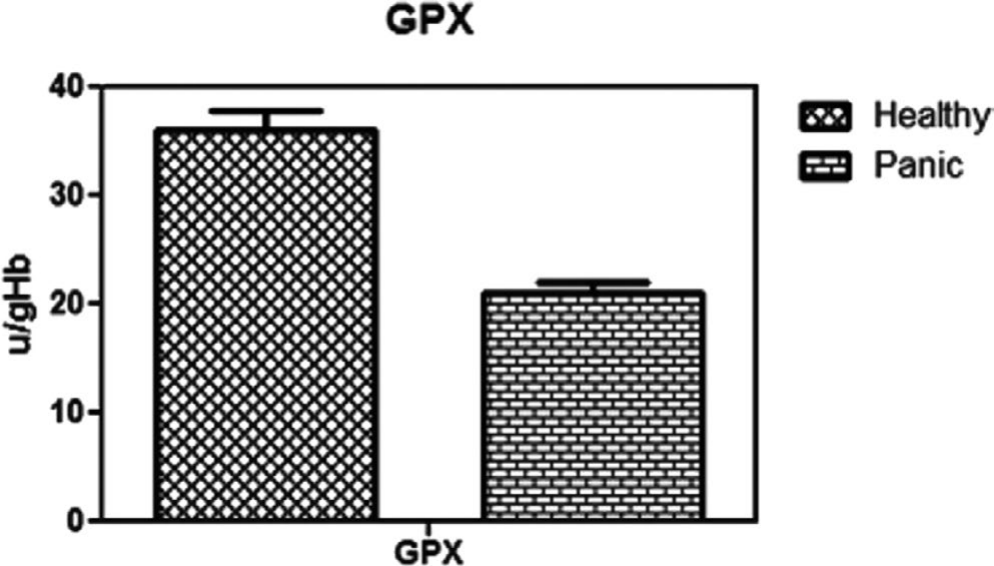
Comparison of GPX between Healthy individuals and panic disorder (PD) patients. It showed GPX activity has a significant decrease in PD patients. The activity of glutathione peroxidase in red blood cells in patients was significantly decreased compared with the group of healthy control (*p* < .01)

**Figure 3:**
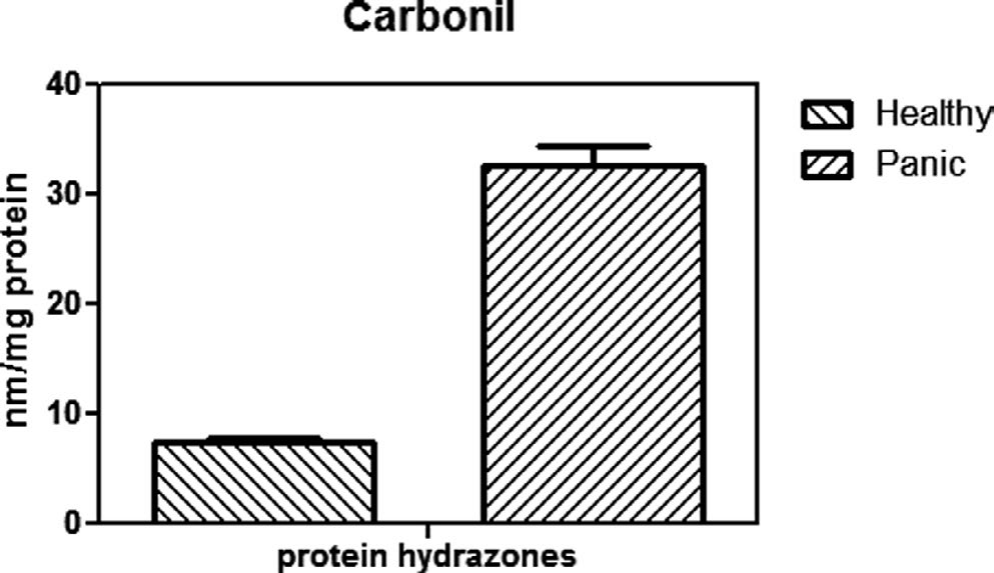
Comparison of Carbonyl groups between Healthy individuals and panic disorder (PD) patients. It showed Carbonyl concentration has a significant increase in PD patients. The concentration of carbonyl groups (hydrazone protein) in patients group was increased significantly compared with healthy individuals (*p* < .001)

**Figure 4:**
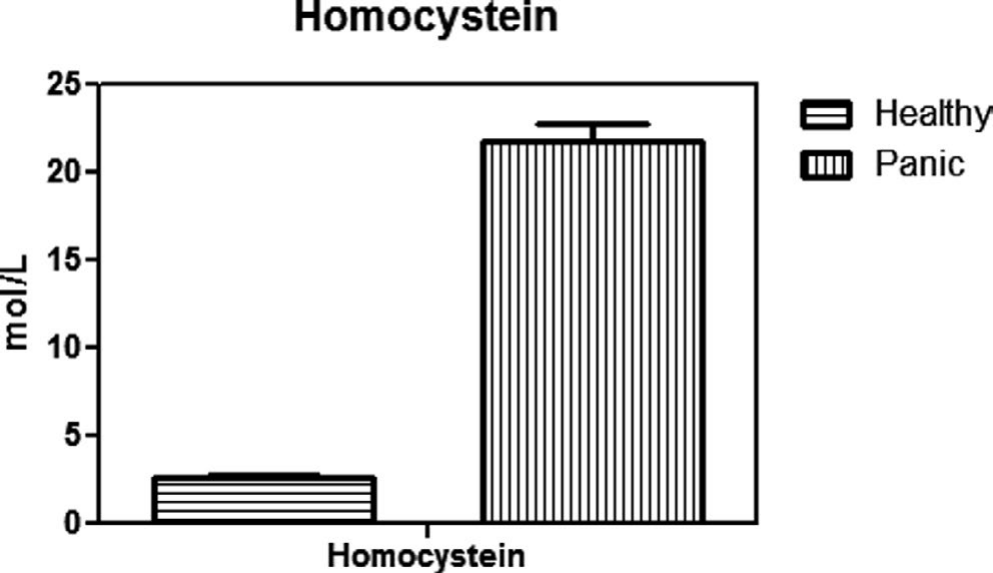
Comparison of Homocysteine between Healthy individuals and panic disorder (PD) patients. It showed Homocysteine concentration has a significant increase in PD patients. Homocysteine levels are significantly higher in PD group than in healthy people (*p* < .05)

As Figure 1 indicates, patients with PD have lower levels of red blood cell glutathione than healthy people, which was statistically significant (*p* < .003). In addition, the activity of glutathione peroxidase in red blood cells in patients was significantly decreased compared with the group of healthy control (Figure 2) (*p* < .01). Figure 3 shows that the concentration of carbonyl groups (hydrazone protein) in patients group was increased significantly compared with healthy individuals (*p* < .001). As it shown in Figure 4, homocysteine levels are significantly higher in PD group than in healthy people. Comparing these changes in gender and age did not show a significant difference (*p* < .05).

## 4 DISCUSSION

The present study showed that serum homocysteine concentrations in patients with PD are significantly different from healthy individuals. Based on other evidence, including increased oxidative stress, these patients are at risk for cardiovascular disease. In our previous study, platelet activation through triggeration by oxidative stress was observed, which indicated a high risk of patients with PD to develop cardiovascular disease (Hamzekolaei et al., 2020).

In Stanger et al. study, which looked at 30 healthy individuals and 30 PD patients, found that in the group of patients, the homocysteine concentrations were clinically in the middle level (>12 µMol/L and <30 µMol), whereas the controls group were within the normal range (Stanger et al., 2004). Relevant studies have previously reported a correlation between homocysteine concentrations and anxiety levels (Pitsavos et al., 2006) and also an association between elevated homocysteine levels and psychological disorders such as stress and anxiety (Atmaca et al., 2005).

Tiemeier et al. (2002) have shown that people with depression have higher levels of homocysteine in their blood. In addition, Chen et al. (2010) reported elevated plasma homocysteine concentrations in the elderly with severe depression. Further, Bjelland et al. (2003) showed that people with depression had higher blood homocysteine levels than healthy individuals. They also observed that levels of depression were directly related to homocysteine concentrations (Bjelland et al., 2003). Another study that looked at homocysteine concentrations in people with bipolar disorder also reported an increase in homocysteine levels (Dittmann et al., 2008).

In this regard, not many studies have been done on homocysteine levels in patients with PD. Meier et al and Yapislar et al showed that patients with PD had high levels of homocysteine in their blood. They also showed that homocysteine levels in these patients were associated with the severity of symptoms (Meier et al., 2010; Yapislar et al., 2012). Normally, the concentration of homocysteine is related to several factors such as age, sex, and hereditary factors.

Researchers have also reported that increased homocysteine levels elevate the anxiety effects by direct neurotoxic effects (Bisschops et al., 2004; Kruman et al., 2000; Sachdev, 2004), reducing the synthesis of neurotransmitters due to decreased and nonrecycling of s‐adenosyl methionine (Bottiglieri et al., 1992, 2000), and/or damage to the vascular wall (Bell et al., 1992; Sachdev et al., 2003). Accordingly, increasing the homocysteine concentration can be expected to elevate the number and frequency of panic attacks (Sawai et al., 2008; Stoney, 1999).

In addition, studies have shown that elevation in homocysteine levels is associated with increased serotonin levels (Ebesunun et al., 2012). Due to the fact that serotonin metabolism disorder is one of the pathogens in panic, the results of the present study are confirmed. In other words, increased serotonin levels elevate its catabolism and as a result the activity of the enzyme monoamine oxidase, which in turn leads to the induction and increase of oxidative stress (Hamzekolaei et al., 2020; Sturza, et al., 2019; Sturza, et al., 2019; Xu et al., 2019), demonstrated in our results by reduced level of glutathione and glutathione peroxidase activists and elevated levels of carbonyl proteins. Our previous study also reported an increase in malondialdehyde levels as an indicator of oxidative stress (Hamzekolaei et al., 2020). Additionally, increased levels of oxidative stress and concomitant increase in homocysteine levels indicate a high risk of cardiovascular disease in panic patients. This issue is explained by Chellappa and Ramaraj (2009) that people with neurological depression were at risk for cardiovascular diseases.

Researchers have also shown that elevated homocysteine levels cause damage and hardening of the vascular wall (Meier et al., 2010). It has been suggested that increased homocysteine can affect endothelial cells and induce prothrombotic state with platelet activation (Hamzekolaei et al., 2020; Pasterkamp et al., 2002). Increased homocysteine levels also induce inflammation, which its effects on atherosclerosis have been reported before (Pasterkamp et al., 2002).

According to what we know today, an increase in homocysteine concentrations can be due to a deficiency of vitamins B6, B12, and folic acid (Medici et al., 2010), alcohol consumption, and lifestyle (Unt et al., 2008). Regarding previous studies and our results, it is suggested that in the treatment of patients with PD, the complications of atherosclerosis should be considered. Therefore, in addition to psychological treatment, vitamin supplements and proper lifestyle as well as diet should be considered for patients to minimize the complications of the disease. In patients with Parkinson's disease, it has been shown that a proper lifestyle combined with regular exercise reduces homocysteine levels (Nascimento et al., 2011).

## 5 CONCLUSION

It is essential to find an effective mechanism to justify the increase in homocysteine levels in patients with PD in order to reduce the side effects of the disease. However, taking vitamin and antioxidant supplements and a healthy lifestyle is recommended to reduce these side effects.

## CONFLICT OF INTEREST

Authors declare that there is no conflict of interest.

## AUTHOR'S CONTRIBUTION

Seyed Shahrokh Aghayan contributed to conceptualization, data curation, and investigation; Asghar Farajzadeh contributed to investigation; Zahra Bagheri‐Hosseinabadi contributed to software; Homeyra Fadaei contributed to methodology and formal analysis; Maryam Yarmohammadi contributed to validation and visualization; Moslem Jafarisani contributed to project administration, writing—original draft, writing—review editing, and supervision.

### Peer Review

The peer review history for this article is available at https://publons.com/publon/10.1002/brb3.1851.

## Data Availability

The data that support the findings of this study are available from the corresponding author upon reasonable request.
